# Ethical Challenges of Big Data in Public Health

**DOI:** 10.1371/journal.pcbi.1003904

**Published:** 2015-02-09

**Authors:** Effy Vayena, Marcel Salathé, Lawrence C. Madoff, John S. Brownstein

**Affiliations:** 1 Institute of Biomedical Ethics, University of Zurich, Zurich, Switzerland; 2 Center for Infectious Disease Dynamics, Department of Biology, Penn State University, University Park, Pennsylvania, United States of America; 3 ProMED (Program for Monitoring Emerging Diseases), International Society for Infectious Diseases and University of Massachusetts Medical School, Worcester, Massachusetts, United States of America; 4 Children’s Hospital Informatics Program, Boston Children’s Hospital, Harvard Medical School, Boston, Massachusetts, United States of America; National Institutes of Health, UNITED STATES

## Introduction

Digital epidemiology, also referred to as digital disease detection (DDD), is motivated by the same objectives as traditional epidemiology. However, DDD focuses on electronic data sources that emerged with the advent of information technology [1–3]. It draws on developments such as the widespread availability of Internet access, the explosive growth in mobile devices, and online sharing platforms, which constantly generate vast amounts of data containing health-related information, even though they are not always collected with public health as an objective. Furthermore, this novel approach builds on the idea that information relevant to public health is now increasingly generated directly by the population through their use of online services, without their necessarily having engaged with the health care system [4, 5]. By utilizing global real-time data, DDD promises accelerated disease outbreak detection, and examples of this enhanced timeliness in detection have already been reported in the literature. The most recent example is the 2014 Ebola virus outbreak in West Africa [6]. Reports of the emerging outbreak were detected by digital surveillance channels in advance of official reports. Furthermore, information gleaned by the various datasets can be used for several epidemiological purposes beyond early detection of disease outbreaks [7, 8], such as the assessment of health behavior and attitudes [4] and pharmacovigilance [9].

This is a nascent field that is developing rapidly [10]. While changes in the ways in which epidemiologic information is obtained, analyzed, and disseminated are likely to result in great social benefits, it is important to recognize and anticipate potential risks and unintended consequences. In this article we identify some of the key ethical challenges associated with DDD activities and outline a framework for addressing them. We argue that it is important to engage with these questions while the field is at an early stage of evolution in order to make ethical awareness integral to its development.

## The Context in Which DDD Operates

DDD operates at the intersection of personal information, public health, and information technologies, and increasingly within the so-called *big data* environment. *Big data* lacks a widely accepted definition. The term has, nevertheless, acquired substantial rhetorical power. We use it here in the sense of very large, complex, and versatile sets of data that are constantly evolving in terms of format and velocity [11]. This dynamic environment generates various ethical challenges that relate not only to the value of health for individuals and societies, but also to individual rights and other moral requirements. In order to spell out these challenges and possible ways of meeting them, it is necessary to take into account the distinctive nature of DDD and the broader context in which it operates. Generally, these distinct features are linked to the methods by which data are generated, the purposes for which they are collected and stored, the kind of information that is inferred by their analysis, and eventually how that information is translated into practice [12]. More specifically, some of these relevant features include those outlined below—namely, the steady growth of digital data, the multifaceted character of big data, and ethical oversight and governance.

### The steady growth of digital data

The amount of data that is generated from activities facilitated by the Internet and mobile technologies is unprecedented. The global number of mobile-cellular subscriptions is close to the world’s population figures, with a total penetration rate of 96%. The mobile-cellular penetration rate in developing countries is 89%, and about 40% of the world’s population is connected to the internet [13]. 82% of the world’s online population uses social media and networks. [14]. More than 40,000 health apps are available, and a new higher-level Internet domain name “health” is about to be released [15, 16]. Not surprisingly, personal data have recently been described as a new asset class with the potential to, among other things, transform health care and global public health [17].

### The multifaceted character of big data

Big data cannot be readily grouped into clearly demarcated functional categories. Depending on how they are queried and combined with other datasets, a given dataset can traverse categories in unpredictable ways. For example, health data can now be extracted from our purchases of everyday goods, our social media exchanges, and our web searches. New data analytics constantly change the kinds of outcomes that become possible. They go beyond early identification of outbreaks and disease patterns to include predictions of the event’s trajectory or likelihood of reoccurrence [18, 19]. These new possibilities render good data governance, which ensures their ethical use, all the more complex.

### Ethical oversight and governance

Public health surveillance and public health research are governed by national and international legislation and guidelines. However, many of these norms were developed in response to very different historical conditions, including technologies that have now been superseded [20]. Such mechanisms may not be appropriate or effective in addressing the new ethical challenges posed by DDD, nor the questions that will be raised if DDD is effectively integrated into standard public health systems. Health research utilizing social media data and other online datasets has already exerted pressure on existing research governance procedures [21].

## Ethical Challenges

Against this background we have identified three clusters of ethical challenges facing DDD that require consideration (Table 1).

**Table 1:** Mapping the ethical issues in digital disease detection.

**Categories**	**Ethical Challenges**	**Specific Examples**	**Values**
Context sensitivity	Differentiating between commercial versus public health uses of data	Is identification permitted? Is consent required for DDD uses? If so, has consent been obtained? Can it be revoked?	Privacy and contextual integrity
	User agreements, terms of service, participatory epidemiology	Are users protected in all contexts irrespective of privacy laws that differ according to jurisdiction?	Transparency
	Global health issues	Are privately collected data open to global public health uses?	Global justice
Nexus of ethics and methodology	Robust methodology: algorithm validation, algorithm recalibration, noise filtering, and feedback mechanisms	False identification of outbreaks and inaccurate predictions of outbreak trajectory	Risk of harm
		Pressure to mobilize public health resources in light of rapidly spreading unvalidated predictions	Fair use of resources
	Data provenance	Awareness about public health uses of personal data (in aggregated form)	Trust, transparency, accountability
Legitimacy requirements	Best practice standards	Is there a shared code of practice amongst all those working on DDD?	Trustworthiness
	Monitoring bodies (policies for ongoing monitoring and action plans for correction of false results)	Is there a mechanism for quick response to inaccuracies about outbreaks?	Trust, transparency, accountability
	Paced integration of DDD to standard surveillance systems	Are there mechanisms for redressing harms caused by DDD activities?	Justice
	Communication to the public (prevent hype)	Management of expectations	Common good

### A. Context sensitivity

At the crux of the debate on the ethics of big data lies a familiar, but formidably complex, question: how can big data be utilized for the common good whilst respecting individual rights and liberties, such as the right to privacy? What are the acceptable trade-offs between individual rights and the common good, and how do we determine the thresholds for such trade-offs? These ethical concerns and the tensions between them are not new to public health research and practice, but now they must be addressed in a new context, with the result that appropriate standards may vary according to the type of big data activity in question.

It is clear that the context of DDD differs in significant ways from other types of big data activity concerned with health. DDD has a public health function, aiming ultimately to improve health at the population level. Public health is a common good from which all individuals benefit and one that is essential to human development and prosperity. There is a clear contrast here with forms of corporate activity that may use the exact same data (i.e., social networking data), but for other purposes, such as advertising. The former aims at fostering a public good (health); the latter at generating a corporate profit. Such differences have important ethical implications. A context-sensitive understanding of ethical obligations may reveal that some data uses that may not be acceptable within corporate activity (e.g., user profiling and data sharing with third parties) may be permissible for public health purposes. Furthermore, societal obligations to foster the common good of public health may generate duties on corporate data collectors to make data available for use in DDD.

Pursuing this line of thought, it is arguable that privacy considerations that apply in standard public health practice will have to be creatively extended and adapted to the case of DDD. This will result in new standards that relate to data from a diverse range of sources, e.g., self-tracking, citizen scientists, social networks, volunteers, or other participatory contexts [22, 23]. Such new standards are urgently needed, especially as greater convergence of datasets becomes possible. An illustration of global activity on this front is the United Nations Global Pulse project [24]. This project explores the concept of data philanthropy whereby public–private partnerships are formed to share data for the public good. Such so-called *data commons*, operating on the basis of clear rules about privacy and codes of conduct, can profoundly affect disease surveillance and public health research more generally.

Another dimension of context relates to global justice. Historically, new health tools have been predominantly used to improve the health of inhabitants of the better-off parts of the world. DDD projects that access global data are often less costly than traditional public health approaches. They could thus offer a potential breakthrough in early disease detection that would benefit communities throughout the world [25, 26]. However, this potential brings moral obligations in its train. This requires not only efforts to detect diseases in poorer parts of the world but also measures to ensure that the way data are collected and processed respect the rights and interests of people from these diverse regions and communities. This raises difficult questions of cultural relativity, such as whether standards of privacy can take different forms in relation to different cultures or whether some minimal core of uniform standards is also justified.

### B. Nexus of ethics and methodology

Robust scientific methodology involves the validation of algorithms, an understanding of confounding, filtering systems for noisy data, managing biases, the selection of appropriate data streams, and so on. Some have expressed skepticism about the role that DDD can play in public health practice given its early state of development [27]. In 2013, when Google Flu Trends overestimated flu prevalence levels in the US, further concerns were raised about the sensitivity of this methodology to the digital environments created by users’ behavior—for example, different uses of search terms [28] from those used to develop the initial algorithm or the distorting influence of searches arising from media coverage of the flu [29, 30].

Methodological robustness is an ethical, not just a scientific, requirement. This is not only because limited resources are wasted on producing defective results or because trust in scientific findings is undermined by misleading or inaccurate findings. There is a further risk of harm to individuals, businesses, or communities if they are falsely identified as affected by an infectious disease. The harm can take many forms, including financial losses, such as a tourist region being falsely identified as the location of a disease outbreak; stigmatization of particular communities, which may adversely affect individual members; and even the infringement of individual freedoms, such as the freedom of movement of an individual falsely identified as a carrier of a particular disease.

The issue of data provenance comes within the remit of ethically sound methodology. Currently published DDD studies and other initiatives have mostly used data that are in the public domain (e.g., Twitter) or that have been contributed by individuals with their explicit consent for use in disease surveillance (flunearyou.org). While in principle data in the public domain are open to being used for public health purposes, what constitutes public domain on the Internet is the subject of lively debate [31]. Especially in the context of data derived from social network interactions, it remains unclear whether users understand in what ways their data can be used and who may access them [32]. Any DDD project will inevitably have to navigate this uncertain environment and so must exercise diligence about data provenance and exhibit transparency about its uses.

### C. Bootstrapping legitimacy

Legitimacy concerns the extent to which DDD is actually ethically justified in imposing the compliance burdens that it does and also the extent to which it is perceived to be ethically justified. In recent years the concept of “global health security” has been mobilized by international organizations, nongovernmental organizations, and national governments to strengthen the legitimacy of systems of disease surveillance both nationally and globally. The idea of human security has been expanded to include health (protection from infectious diseases and other health hazards), augmenting state responsibilities to provide appropriate safeguards. The revised International Health Regulations [33], which set out a global legal framework for disease detection and response, are premised on the understanding that in our globalized world diseases spread rapidly and therefore on the need for the timely notification of any public health threat of potentially international significance. They also recognize the importance of information gathering from various sources, including unofficial or informal ones, whilst also requiring that the validity of such information be verified [34]. This creates a legitimate space for DDD activities because they are precisely responses to both the accelerated detection and the global nature of the spread of disease.

However, even if ethical arguments already justify the DDD enterprise, they only serve as a starting point. DDD will have to build its own legitimacy over time as an integral part of its approach. This means that the issues under categories A and B have to be constantly engaged with thorough processes that bootstrap DDD’s legitimacy, so it is continuously self-generating and enhanced over time. So, for example, it is not enough simply to appeal to the great contribution that DDD stands to make to the common good of public health. It is important that this contribution is made in certain ways rather than others, through transparent procedures that are worthy of engendering trust among those individuals whose data are used in DDD.

Current regulatory and ethical oversight mechanisms are ill-equipped to address the entire spectrum of DDD-type activities. The distinction between public health and public health research has long been considered a problematic one, and this is even more evident in the DDD context. Consider an analogy with participant-led biomedical research—a growing movement of people collecting data about themselves and conducting various forms of research in large groups. Either such activities fall through the cracks of the existing oversight mechanisms or else, if they do not, those mechanisms impose inappropriate burdens upon them [35, 36]. Participatory approaches to disease surveillance confront similar challenges. Individuals report on disease symptoms on online platforms, (e.g., flunearyou.org) which enables them to contribute to the common good of disease surveillance and often to receive feedback about disease prevalence in their area [37]. This active participation potentially empowers individuals and democratizes the process of scientific discovery. However, data (personally identifiable information, geolocation, etc.) that are collected for DDD purposes need to be governed in ways that minimize the risk of harm to participants. For example, if individuals take personal risks in order to report events of public health importance (i.e., a farmer reporting avian flu at risk of losing his flock), those risks should be mitigated by appropriate policies (e.g., compensation) that acknowledge the societal contribution and the local/personal costs.

For the purposes of ensuring its legitimacy, DDD must develop internal mechanisms such as its own best-practice standards, including monitoring boards with the concrete mandate to ensure that risks and costs to individuals and communities are proportional to benefits. Such boards should also be empowered to negotiate compensation schemes for harms that have been suffered. As in standard public health practice individuals may be adversely affected by a practice that aims to secure the health of the population. However, this laudable goal does not remove the obligation to respect individual rights and dignity in its pursuit. Neither of these standards are to be equated with an automatic insistence on individual consent. Instead, they consist of distinct individual entitlements, of the sort set out in the Universal Declaration of Human Rights, and the inherent value in all human beings, which underlies them.

## Conclusions

The emergence of DDD promises tangible global public health benefits, but these are accompanied by significant ethical challenges. While some of the challenges are inherent to public health practice and are only accentuated by the use of digital tools, others are specific to this approach and largely unprecedented. They span a wide spectrum, ranging from risks to individual rights, such as privacy and concerns about autonomy, to individuals’ obligations to contribute to the common good and the demands of transparency and trust. We have grouped these concerns under the headings of context sensitivity, nexus of ethics and methodology, and bootstrapping legitimacy. It is vital that engagement with these challenges comes to be seen as part of the development of DDD itself, not as some extrinsic constraint. We intend this paper to be a contribution to the development of a more comprehensive and concrete ethical framework for DDD, one that will enable DDD to find an ethical pathway to realizing its great potential for public health.
